# Evaluation of the Endothelin Receptor Antagonists Ambrisentan, Bosentan, Macitentan, and Sitaxsentan as Hepatobiliary Transporter Inhibitors and Substrates in Sandwich-Cultured Human Hepatocytes

**DOI:** 10.1371/journal.pone.0087548

**Published:** 2014-01-30

**Authors:** Eve-Irene Lepist, Hunter Gillies, William Smith, Jia Hao, Cassandra Hubert, Robert L. St. Claire, Kenneth R. Brouwer, Adrian S. Ray

**Affiliations:** 1 Gilead Sciences, Inc., Foster City, California, United States of America; 2 Qualyst Transporter Solutions, LLC, Durham, North Carolina, United States of America; University of Cambridge, United Kingdom

## Abstract

**Background:**

Inhibition of the transporter-mediated hepatobiliary elimination of bile salts is a putative mechanism for liver toxicity observed with some endothelin receptor antagonists (ERAs).

**Methods:**

Sandwich-cultured human hepatocytes were used to study the hepatobiliary distribution and accumulation of exogenous taurocholate, ERAs and endogenous bile acids. The molecular mechanisms for findings in hepatocytes or clinical observations were further explored using either vesicular assays (efflux transporters) or transfected cell-lines (uptake transporters). Inhibition constants (IC_50_) were measured for the human hepatobiliary transporters bile salt export pump (BSEP), sodium taurocholate cotransporting polypeptide (NTCP), multidrug resistance protein 2 (MRP2), P-glycoprotein (Pgp), breast cancer resistance protein (BCRP), organic anion-transporting polypeptide 1B1 (OATP1B1) and OATP1B3.

**Results:**

The ERAs showed dose-dependent reductions in exogenous taurocholate cellular accumulation in human hepatocytes, with macitentan having the greatest effect. Consistent with their effects on bile acids, the ERAs inhibited bile transporters. IC_50_ values for OATP1B1 and OATP1B3 ranged from 2 µM for macitentan to 47 µM for ambrisentan. Macitentan and bosentan also inhibited NTCP with IC_50_ values of 10 and 36 µM, respectively. Similar to previously reported findings with sitaxsentan, BSEP inhibition was observed for bosentan and macitentan with IC_50_ values of 42 and 12 µM, respectively. In contrast, ambrisentan showed little or no inhibition of these transporters. Other transporters tested were weakly inhibited by the ERAs. Accumulation in hepatocytes was also a factor in the effects on bile transport. Macitentan demonstrated the greatest accumulation in human hepatocytes (∼100x) followed by sitaxsentan (∼40x), bosentan (∼20x) and ambrisentan (∼2x).

**Conclusions:**

Significant differences in the inhibition of hepatic transporters were observed between the evaluated ERAs *in vitro*. Macitentan had the highest level of cellular accumulation and caused the greatest effects on bile acid distribution in human hepatocytes followed by sitaxsentan and bosentan. Ambrisentan showed a low potential to affect bile acids.

## Introduction

Pulmonary arterial hypertension (PAH) is a progressive and fatal disease, characterized by increasing pulmonary vascular resistance leading to right ventricular failure and premature death [1]. Current treatment for PAH targets one or more of three central biological pathways involved in the pathogenesis of the disease: the prostacyclin, nitric oxide and endothelin pathways [1], [2]. Endothelin-1 (ET-1) is a potent vasoconstrictor peptide thought to play a critical role in the pathogenesis and progression of PAH [3]–[5]. The biological effects of ET-1 are mediated through the endothelin receptor subtype A (ET_A_) and endothelin receptor subtype B (ET_B_). Endothelin receptor antagonists (ERAs) developed to date for the treatment of PAH include ambrisentan, bosentan, and sitaxsentan. Another ERA, macitentan, has recently been approved by the FDA. Of these, ambrisentan and sitaxsentan are ET_A_-selective ERAs while bosentan and macitentan have mixed activity against both ET_A_ and ET_B_ receptors. Chemically, ambrisentan is propanoic acid-based while bosentan, sitaxsentan, and macitentan are sulfonamide-based.

In PAH clinical trials, the ERA class of therapeutics has been shown to improve exercise capacity, improve functional capacity, and delay clinical worsening [6]–[12]. Despite these clinical benefits, bosentan and sitaxsentan have been associated with evidence of liver toxicity as indicated by elevated serum aminotransferase levels [7], [13]. In fact, sitaxsentan was recently withdrawn from the worldwide market due to hepatotoxicity, with several cases of idiosyncratic and sometimes fatal liver toxicity [14]. Conversely, evidence to date suggests that ambrisentan is associated with a low risk of hepatic injury [11], [12]. In the pivotal ARIES studies, none of the patients randomized to ambrisentan (n = 261) experienced serum aminotransferase concentrations >3 times the upper limit of normal (ULN) as compared to several patients receiving placebo [11]. This pattern continued in the long-term extension study (ARIES-E), with an incidence of elevated aminotransferase >3 times ULN of 1.8% over a mean follow-up of 1.4 years [12]. While the biological reasons for these observed differences in incidence of liver abnormalities between these ERAs are unclear, it is possible that there is a link between chemical structure (i.e. propanoic acid vs. sulfonamide), endothelin receptor subtype selectivity and/or differences in hepatocyte uptake, efflux, and accumulation. Notably, while ET_A_ is the main receptor subtype in smooth muscle cells, ET_B_ receptors are the predominate subtype in human liver cells and ET_B_ blockade has recently been linked to portal sinusoid constriction, suggesting that ET_B_ antagonism in the liver may contribute to the hepatotoxicity seen with nonselective ERAs [15], [16].

In the phase 3 SERAPHIN study, PAH patients receiving macitentan therapy for up to 103.9 weeks had a significantly reduced risk of morbidity and mortality [17]. In preclinical compound screening, macitentan was selected for its lipophilic properties and its low tendency to increase circulating bile salts when administered intravenously in rats [18]. In early testing in humans, short-term dosing with macitentan did not appear to result in any dose-dependent alterations in circulating aminotransferases [18]. Preliminary data from the SERAPHIN study indicated that the incidence of serum aminotransferase elevations >3X ULN was 3.4% to 3.6% for macitentan patients as compared to 4.5% in placebo patients [17], [19]. However, elevations >8X ULN were ∼5-fold greater on macitentan (2.1%) compared to placebo (0.4%) [19].

**Figure 1:**
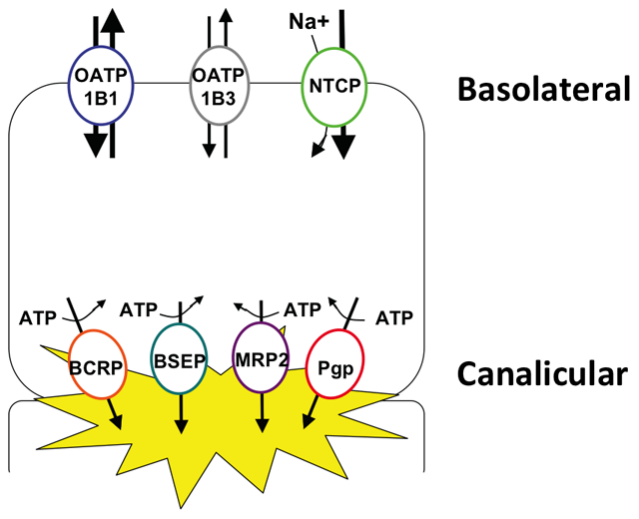
Depiction of hepatocyte basolateral and canalicular transport proteins.

Perturbations in hepatobiliary elimination of bile salts has emerged as a leading hypothesis for the mechanism of liver toxicity observed with some ERAs [20]–[26]. Inhibition of hepatic transporters is the net effect of a complex interplay between intracellular accumulation, transporter inhibition, metabolism and clearance. For this study, we used sandwich-cultured human hepatocytes in order to best recapitulate these processes *in vitro*. Hepatocyte uptake of bile acids is mediated primarily by the basolateral Na^+^-taurocholate cotransporting polypeptide (NTCP) transporters with additional support by organic anion-transporting polypeptides (OATPs) while efflux into the bile canaliculi is mediated by the bile salt export pump (BSEP) and multidrug resistance-associated protein 2 (MRP2) (Figure 1). Alterations in the activity of these proteins can lead to hepatic bile acid accumulation and liver injury [20], [27], [28]. Bosentan has been shown to inhibit BSEP and MRP2, an effect that has been suggested to contribute to the hepatotoxicity seen with this ERA [20], [23]. Similarly, sitaxsentan has been shown to inhibit NTCP, OATP and BSEP [29].

In the last few years, BSEP inhibition has emerged as a probable mechanism for the development of drug-induced liver injury [21], [22], [24]–[26], [30]. In transgenic mice, inactivation of BSEP results in a mild, persistent hepatic cholestasis which progresses towards severe cholestasis with high mortality upon cholic acid feeding [21], [22]. In a comprehensive study of more than 200 clinical compounds by Morgan et al., a strong correlation was observed between BSEP inhibition and reported evidence of liver toxicity [26]. In a follow-up study of >600 marketed or withdrawn drugs, those compounds with an estimated steady-state concentration/BSEP IC_50_ ratio ≥0.1 had an almost 100% correlation with some evidence of liver injury in humans [30]. Finally, hereditary BSEP deficiency leads to end-stage liver disease in humans [31]–[33]. Perhaps the most compelling evidence for a temporal relationship between BSEP inhibition and cholestatic liver injury comes from three cases of patients with hereditary BSEP deficiency that were treated by liver transplantation but re-developed cholestatic dysfunction post-transplant. In these individuals, recurrence of disease correlated with the presence of BSEP antibodies that potently inhibited bile salt transport. Consistent with the causative role of these antibodies, the cholestatic dysfunction in these patients was reversed by immunosuppressive therapy [25].

The objective of this study, therefore, was to investigate possible mechanisms of clinical hepatotoxicity and the relative potential to cause cholestatic liver injury for the ERAs ambrisentan, bosentan, sitaxsentan, and macitentan. Hepatocellular accumulation and efflux of the ERAs were evaluated in sandwich-cultured human hepatocytes, as were their effects on bile acid transport. The mechanism for observations in hepatocytes were further studied by assessing the potential for ERAs to inhibit key hepatic transporters *in vitro* using membrane vesicles or transfected cell lines and model substrates.

## Materials and Methods

### Chemicals

Test ERAs (ambrisentan, bosentan, macitentan, and sitaxsentan) were synthesized by Gilead Sciences (Foster City, CA) and stored at −20°C until use. DMSO stock solutions (100 mM) were prepared the day of the study and aliquotted into small volumes in glass vials for long-term storage at −20°C.

### Human Hepatocyte Isolation and Culture

Sandwich-cultured human hepatocytes were obtained from Celsis/IVT (Baltimore, MD) or Life Technologies (Durham, NC). Donors were male (n = 3) and female (n = 1) between the ages of 31 and 62 years old. Hepatic uptake, biliary excretion and *in vitro* biliary clearance (Cl_b_) were determined using the B-CLEAR® hepatocyte sandwich culture model as described by Liu et al., 1999 [34]. In this system, hepatocytes form functional bile canalicular networks while maintaining the expression and function of key uptake and efflux transporters. The canalicular system of bile pockets remains separate from the cellular culture media due to tight junctions between cells. The integrity of these tight junctions is maintained in the presence of calcium (Plus (+) buffer) while in the absence of calcium (Minus (−) buffer), these tight junctions open and release the contents of the canalicular network into the media.

### ERA Effect on Hepatobiliary Disposition of d_8_-Taurocholate

Pre-incubation solutions containing the ERAs (100 µM) or rifamycin-SV (100 µM, control inhibitor) were prepared in Plus (+) Buffer and Minus (−) Buffers. A dose solution containing 2.5 µM d_8_-taurocholic acid (d_8_-TCA) was prepared in Plus (+) Buffer. Co-incubation solutions were prepared by combining d_8_-TCA dose solution and ERA or rifamycin-SV. Hepatocytes were washed and then conditioned for 10 min in warm Plus (+) or Minus (−) buffer with and without ERA or rifamycin-SV. Pre-incubation solutions were then removed and replaced with dose or co-incubation solutions and incubated for 10 min at 37°C. Following this 10 minute incubation, the dose or co-incubation solutions were removed and the cells were then washed three times with ice-cold Plus (+) buffer. The plates were then frozen at −80°C until processed for bioanalysis.

### Transporter Inhibition Studies

The cellular assays and experimental conditions for the transporter inhibition studies are summarized in Table S1. Chinese Hamster Ovary (CHO) cells were obtained from Professor B. Stieger’s laboratory at University of Zurich, Zurich, Switzerland. Madin-Darby Canine Kidney strain II (MDCKII) cells were obtained from Nederlands Kanker Instituut (NKI), Amsterdam, Netherlands. NTCP-CHO cells, BSEP and MRP2 membrane vesicles were developed and validated at Solvo Biotechnology, Budaors, Hungary.

CHO cells, either wild type or transfected with the genes encoding human NTCP, OATP1B1 and OATP1B3, were maintained in Dulbecco’s Modification of Eagle’s Medium (DMEM) containing 1,000 mg/L D-glucose, L-glutamine, 25 mM HEPES buffer, 110 mg/L sodium pyruvate, 1% Penicillin/Streptomycin, 10% fetal bovine serum (FBS), 0.05 mg/mL L proline and 0.5 mg/mL of geneticin G-418. Cells were maintained in incubators set at 37°C, 90% humidity and 5% CO_2_. OATP1B1 and OATP1B3 over-expressing cells were seeded in BioCoat Poly-D-Lysine coated 96-well black cell culture plates with clear bottoms at a density of 1×10^5^ cells/well. Sodium butyrate (10 mM) was added to the OATP1B1 and OATP1B3 cells once seeded to increase the protein expression level and the cells were grown to confluence overnight. The assay buffer contained 142 mM NaCl, 5 mM KCl, 1 mM KH_2_PO_4_, 1.2 mM MgSO_4_, 1.5 mM CaCl_2_, 5 mM Glucose and 12.5 mM HEPES (pH 7.4). After removal of the media and before adding test compounds, the cells were washed twice with 37°C assay buffer followed by a 0.5 h pre-incubation with assay buffer. Test compounds were diluted in assay buffer containing 2 µM Fluo 3 and pre-incubated with cells for 1 h. Following removal of assay buffer containing Fluo 3 and test compound, cells were washed 3 times with 200 µl of ice cold assay buffer and then lysed at room temperature for 15 min in a lysis buffer containing 0.05% SDS in a 1 mM CaCl_2_ solution. Wells were analyzed for Fluo 3 fluorescence at an excitation of 485 nm and emission of 530 nm. Inhibition of NTCP was studied using similar conditions to those described for OATP1B1 and OATP1B3 except that inhibition of the uptake ^3^H taurocholate into transfected CHO cells was monitored using radioactive scintillation counting.

MDCKII cells were maintained in DMEM with sodium pyruvate, Glutmax, 1% Penicillin/Streptomycin and 10% FBS in an incubator set at 37°C, 90% humidity and 5% CO_2_. MDCKII cells were seeded in 96-well black cell culture plates with clear bottoms at a density of 5×10^4^ cells/well for Pgp and 2×10^4^ cells/well for BCRP and grown to confluence. For the Pgp assay, test compounds were serially diluted in DMSO and then added into in cell culture medium (without FBS) containing 10 µM Calcein AM and incubated for 1 h. Following the removal of media containing Calcein AM and test compound, cells were washed five times with 1 M phosphate buffered saline containing magnesium and calcium (PBS). Wells were analyzed for Calcein AM fluorescence at an excitation of 494 nm and an emission of 517 nm. For the BCRP assay, test compounds were serially diluted DMSO and then spiked in cell culture medium (without FBS) containing 1 µM pheophorbide a (PhA) and incubated for 18 hours with MDCKII-ABCG2 cells. Following the removal of media containing PhA and test compound, cells were then washed five times with PBS. Wells were analyzed for PhA fluorescence at an excitation of 415 nm and an emission of 675 nm.

BSEP and MRP2 inhibition was studied in membrane vesicles isolated from sf9 insect cells overexpressing the respective transporters. Vesicular transport inhibition assays used test compounds incubated with membrane vesicle preparations (total protein: 50 µg/well) and probe substrates, taurocholate (2 µM) for BSEP or Estradiol-17-beta-glucuronide (0.2 µM) for MRP2, in the absence or presence of ATP. Reaction mixtures were preincubated for 15 min at 37°C. Reactions were started by the addition of 25 µL of 12 mM MgATP or assay buffer (for background controls), preincubated separately. Reactions were stopped after 5 min by the addition of 200 µL of ice-cold washing buffer and immediate filtration via glass fiber filters mounted to a 96-well plate (filter plate). The filters were washed, dried and the amount of substrate inside the filtered vesicles determined by liquid scintillation. Cyclosporin A (20 µM) or benzbromarone (100 µM), for BSEP or MRP2, respectively, were used as positive control inhibitors. Control membranes lacking transporter expression were used as negative control.

All assays were performed in duplicate. IC_50_ was defined as the test article concentration needed to inhibit the maximal transporter specific accumulation by 50%. IC_50_ values were calculated using non-linear fitting of % inhibition versus concentration to a sigmoidal curve with a variable Hill Coefficient using GraphPad Prism 5 (GraphPad Software Inc., San Diego, CA).

### Hepatic Disposition of ERAs

Incubation solutions containing ERA (1, 10 and 100 µM) were prepared in Plus (+) buffer. Cell culture medium was removed from the wells and the cells were washed twice with Plus (+) or Minus (−) buffer. The wash solutions were then removed and replaced with fresh Plus (+) or Minus (−) buffer. The cells were conditioned for 10 min at 37°C. After the 10 min exposure, the buffer solutions were removed and replaced with the incubation solutions and incubated for 10 min at 37°C. Following 10 min incubation, the incubation solutions were removed and the cells were then washed three times with ice-cold Plus (+) Buffer. The plates were frozen at −80°C until processed for bioanalysis.

The cellular uptake of bosentan and macitentan was determined in fresh human hepatocytes purchased from Celsis/IVT (Baltimore, MD) or Life Technologies (Foster City, CA). Cells were pre-incubated in Krebs-Henseleit Buffer containing 5% bovine serum albumin for 30 min at 37°C prior to assay. Cells were then co-incubated in the presence and absence of an inhibitor cocktail containing 40 µM rifampicin and 5 µM of cyclosporin A for 15 min. Test compounds bosentan and macitentan were diluted to 3 fold of final target concentration in the same assay buffer and equilibrated at 37°C for 30 min. The uptake assay was initiated by adding 50 µL of test compound solution to the 48-well plate containing 100 µL cell suspension, mixed and incubated at 37°C for 30, 60 and 90 sec. Final cell density was 2×10^6^ cells/mL with a final test compound concentration of 300 nM. The reaction mixture was overlaid onto pre-prepared microcentrifuge tubes containing 100 µL of 2 N NaOH (bottom layer) and 100 µL of filtration oil (middle layer; 74.5∶25.5 silicon oil:mineral oil mix) followed by centrifugation at 13,000 g for 30 sec. The samples were allowed to sit at room temperature for 2 h and then frozen at −80°C. The microcentrifuge tubes were then cut and the bottom layer containing the cell lysate collected. HCl solution was added to neutralize the solution. Samples were extracted with organic solvents for analysis by liquid chromatography coupled to tandem mass spectrometry (LC/MS/MS).

### Effect of ERAs on Endogenous Bile Acids

Incubation solutions containing ERA (1, 10 and 100 µM) were prepared in cell culture medium. On the sixth day of culturing of the sandwich-cultured hepatocytes, cell culture medium was removed from the wells and incubation solutions containing the test ERAs were added to the cells. The cells were incubated with ERA solution for 24 h at 37°C. After the exposure, the incubation solutions were removed and the cells were rinsed with Plus (+) or Minus (−) buffer. The buffer solutions were then removed and the cells were incubated with fresh Plus (+) or Minus (−) buffer for 5 min at 37°C. Following this 5 minute incubation, the buffer solutions were collected and any remaining buffers were removed. The cells were then washed three times with ice-cold Plus (+) buffer and the plates were frozen at −80°C until processed for bioanalysis.

### Bioanalysis

Study samples were stored in their original 24-well plates at −80°C until prepared for analysis. A volume of 500 µL of cell lysis solution (70∶30 methanol:water) containing 25 nM internal standard was added to each well and mixed for 20 min of mixing. The volume in each well was then transferred to a 96-well filter plate stacked on a Greiner deep well collection plate. The filter plate/collection plate was then centrifuged at 2000 rcf for 5 min. The filtrate was then evaporated to dryness under nitrogen. Samples were reconstituted in 200 µL of sample diluent (60∶40 methanol:10 mM ammonium acetate, native pH), sealed, and placed on a plate shaker for 15 min. These samples were then transferred to a Whatman 96-well 0.45 µm PVDF filter plate stacked on a Costar 3956 plate (HPLC injection plate), centrifuged at 2000 rcf for 2–3 min, and sealed with a silicon cap mat prior to LC-MS/MS analysis. d_8_-TCA standard spiking solutions were prepared in 50∶50 methanol:water. For a given lysate standard or control, 10 µL was added to each well of a previously frozen analytical plate. Standards representing 0.5–100 pmoles/well and QC’s at the LLQ and ULQ were prepared. These lysate standards and controls were then further processed as described above for unknown study samples. Prepared samples were filtered and analyzed by LC-MS/MS using a Shimadzu binary HPLC system (Columbia, MD) and tandem mass spectrometry using Thermo Electron TSQ® Quantum Discovery MAX™ (Waltham, MA) with an Ion Max ESI source operating in negative ion electrospray ionization mode. Results for unknowns and QCs were calculated using a standard curve based on an internal standard processing method.

### Data Analysis

Data calculations were performed using Microsoft Excel, 2010 with all values reported as mean ± standard deviation (SD). Statistical comparisons across ERA treatments were performed using analysis of variance (ANOVA) with Fisher’s PLSD post hoc analysis (Statview 5.0, SAS Institute, Cary NC); statistical significance was considered to be P<0.05. All mass values were normalized to mg protein by taking the average protein mass in the taurocholate plate determined using a BCA protein assay (Pierce Biotechnology, Rockford, IL). Cellular accumulation determined in Minus (−) buffer (Accumulation _Minus (−) Buffer_) represents the total mass of analyte inside the hepatocyte at the end of the incubation time period. Total accumulation determined in Plus (+) buffer (Accumulation _Plus (+) Buffer_) represents the total mass of compound taken up and excreted (Cells + Bile). The biliary excretion index (BEI) was calculated according to the following equation:
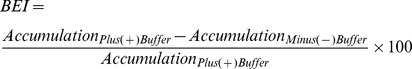



The *in vitro* biliary clearance (Cl_b_) was determined using the equation:
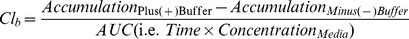



Cl_b_ was scaled to body weight using the factor of 2962 mg protein/kg body mass for sandwich-cultured human hepatocytes (Qualyst, Technical Application Bulletin, TAB Biol 007).

## Results

### ERA Effect on Hepatobiliary Disposition of d_8_-Taurocholate

**Figure 2:**
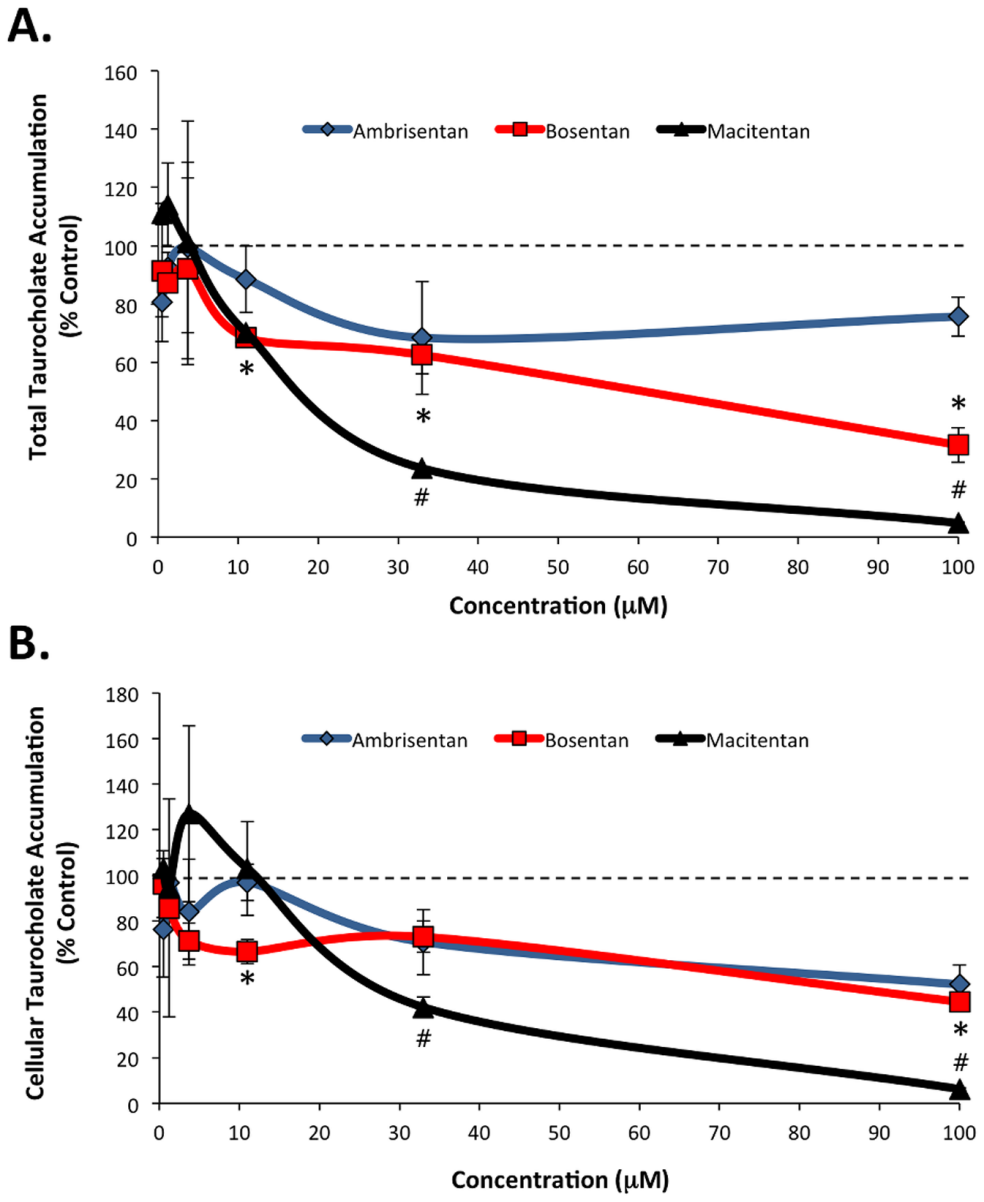
d_8_-Taurocholate (d_8_-TCA) total (A) and cellular (B) accumulation in sandwich-cultured human hepatocytes exposed to ambrisentan, bosentan and macitentan. Bosentan and macitentan treatment resulted in a dose-dependent reduction in total accumulation of d_8_-TCA. Ambrisentan, bosentan and macitentan treatment each resulted in a dose-dependent reduction in cellular accumulation of d_8_-TCA. Data presented as mean (±SD) expressed as percent of control treated; n = 3 donors; *P<0.05 bosentan vs. control; # P<0.05 macitentan vs. control.

In sandwich-cultured hepatocytes, the hepatobiliary disposition of d_8_-TCA is dependent on the NTCP and OATP influx transporters and the BSEP efflux transporter (Figure 1). For the untreated control samples, the mean total accumulation was 226.0±76.0 pmol/mg with a mean cellular accumulation of 58.1±22.7 pmol/mg. The BEI and Cl_b_ for the untreated controls were 74.7% and 19.9±7.8 mL/min/kg, respectively. In the presence of the control inhibitor rifamycin-SV, BEI and Cl_b_ were reduced to 57.1% and 4.5±2.1 mL/min/kg, respectively. Dose-dependent reductions in d_8_-TCA total accumulation were observed for bosentan and macitentan, with total accumulation values (% control) of 68.6±1.5%, 62.6±6.6%, and 31.7±5.9% in the presence of 11, 33, and 100 µM bosentan, respectively and total accumulation values of 23.6±0.5% and 4.8±0.2% for 33 and 100 µM macitentan, respectively (all P<0.05 compared to control) (Figure 2A).

Significant reductions compared to control were also observed in d_8_-TCA cellular accumulation (% control) in the presence of 11 and 100 µM bosentan (66.5±5.3% and 44.5±3.6%, respectively; P<0.05) and for 33 and 100 µM macitentan (42.1±4.5% and 6.2±0.5%, respectively P<0.05) (Figure 2B). While ambrisentan treatment also reduced d_8_-TCA cellular accumulation at higher concentrations (71% at 33 µM and 52% at 100 µM), these effects were not statistically significant. Together, these data suggest that each of the tested ERAs alter NTCP/OATP-mediated uptake of d_8_-TCA into hepatocytes to varying degrees, with macitentan having the largest effect.

**Figure 3:**
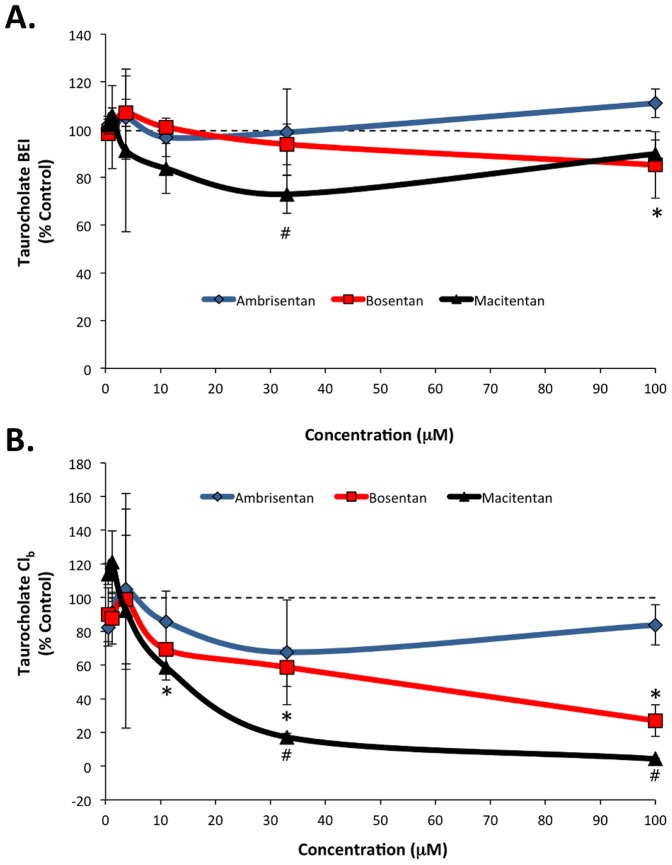
d_8_-Taurocholate (d_8_-TCA) biliary efflux (A) and clearance (B) in sandwich-cultured human hepatocytes exposed to ambrisentan, bosentan, and macitentan. The biliary excretion index (BEI) of d_8_-TCA was largely unaffected by the test ERAs. Bosentan and macitentan treatment resulted in dose-dependent reductions in biliary clearance (Cl_b_) of d_8_-TCA. Data presented as mean (±SD) expressed as percent of control treated; n = 3 donors; *P<0.05 bosentan vs. control; #P<0.05 macitentan vs. control.

The efflux of d_8_-TCA mediated by BSEP was largely unaffected by each of the ERAs, though exposure to 33 µM macitentan and 100 µM bosentan each resulted in slight, but statistically significant, reductions as compared to control (72.9±8.0% and 85.2±13.9%, respectively) (Figure 3A). Analysis of *in vitro* biliary clearance (Cl_b_) indicated dose-dependent reductions in Cl_b_ of d_8_-TCA in treatments compared to control with bosentan (11 µM, 69.2±0.1%; 33 µM, 58.7±11.4%; 100 µM, 27.0±9.3% [all P<0.05]) and macitentan (33 µM, 17.2±2.3%; 100 µM 4.3±0.5% [all P<0.05]) (Figure 3B).

### Inhibition of Hepatic Transporters

To explore the possible molecular mechanisms for the results observed in the human sandwich-cultured hepatocytes, inhibition constants (IC_50_) were measured for BSEP, NTCP, MRP2, Pgp, BCRP, OATP1B1 and OATP1B3; these results are summarized in Table 1. For each assay, the positive control inhibitors for each transporter showed >85% inhibition. Ambrisentan weakly inhibited OATP1B1 and OATP1B3, with IC_50_ values of 47.0 and 44.6 µM, respectively. At up to the highest concentration tested (100 µM), ambrisentan did not inhibit NTCP, BCRP, BSEP or Pgp (IC_50_>100 µM). Bosentan and macitentan more potently inhibited OATP1B1 (5.0 and 2.0 µM, respectively) and OATP1B3 (5.2 and 2.1 µM, respectively). Bosentan inhibited NTCP and BSEP with IC_50_ values of 35.6 and 42.1 µM, respectively, and showed no inhibition of BCRP or Pgp (IC_50_>100 µM). Similarly, sitaxsentan has under similar conditions shown no inhibition of BCRP or Pgp (IC_50_>100 µM) and inhibited BSEP with an IC_50_ of 25 µM [35]. Macitentan inhibited BSEP, NTCP, BCRP and Pgp with IC_50_ values of 11.9, 9.8, 75.0, and 64.0 µM, respectively. The ERAs showed weak or no inhibition of MRP2, with IC_50_ values from ∼75 to >100 µM.

**Table 1:** Effect of Ambrisentan, Bosentan, Sitaxsentan, and Macitentan on Hepatic Uptake and Efflux Transporters.

Transporter	Ambrisentan	Bosentan	Sitaxsentan	Macitentan
	(IC_50_)	(IC_50_)	(IC_50_)	(IC_50_)
OATP1B1^a^	47.0±21.3	5.0±2.0	ND^c^	2.0±0.3
OATP1B3^a^	44.6±23.8	5.2±2.1	ND^c^	2.1±0.3
NTCP^a^	>100	35.6±4.9	ND^c^	9.8±1.5
BCRP^a^	>100	>100	>100^c^	75±37
BSEP^a^	>100	42.1±14.6	25^c^	11.9±1.1
MRP2^b^	∼75	>100	>100^c^	>100
Pgp^a^	>100	>100	>100^c^	64±15

a Data presented as mean ± standard deviation for 3 independent studies performed in duplicate;

b Data presented for a single experiment preformed in duplicate;

c Data previously reported [35]. Ambrisentan, bosentan, and macitentan were tested in concentrations ranging from 0.14–100 µM.

ND = not determined.

### Hepatobiliary Disposition of ERAs

**Figure 4:**
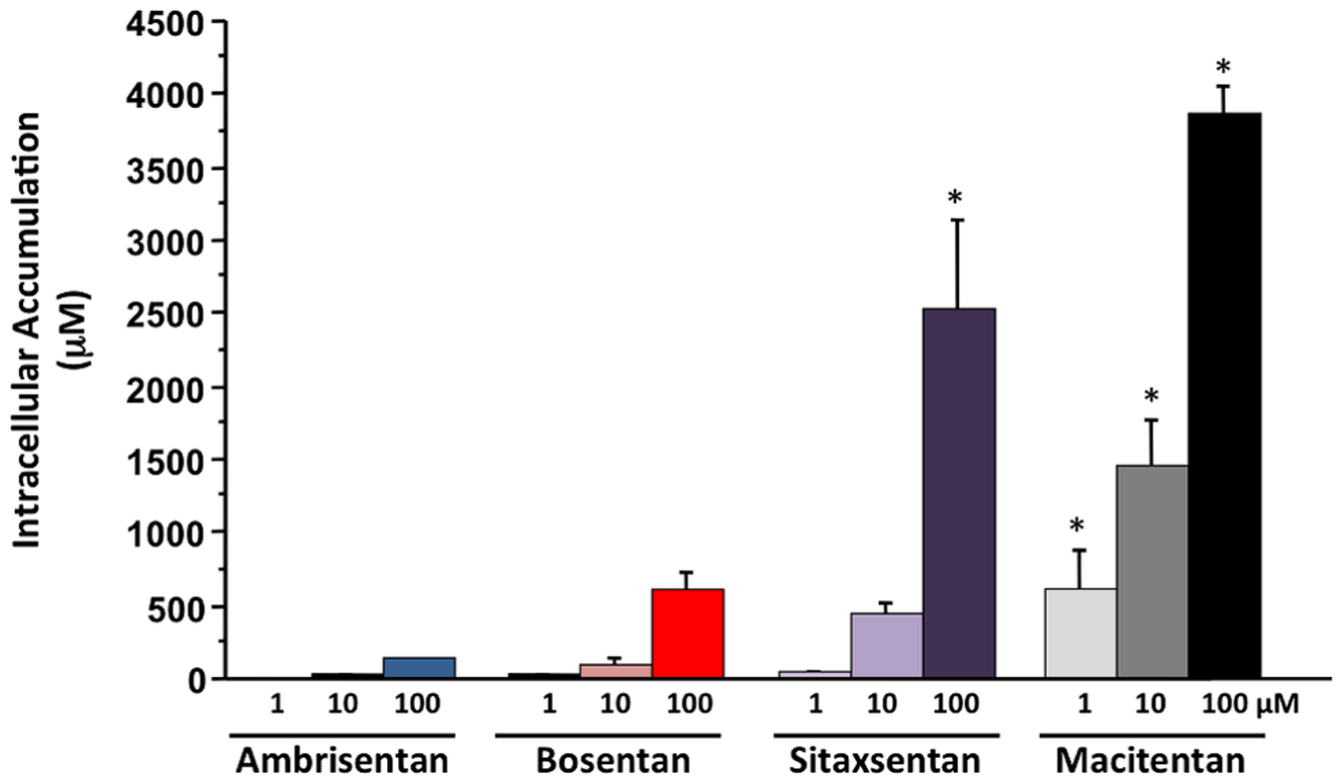
Dose-dependent intracellular accumulation of test ERAs in sandwich-cultured human hepatocytes. Ambrisentan displayed the lowest intracellular accumulation followed by bosentan, sitaxsentan, and macitentan. Data are presented as mean (±SD) micromolar (µM) concentration; n = 3 donors; *P<0.05 vs. corresponding intracellular accumulation value for ambrisentan at the same test concentration.

The intracellular concentration is the primary driving force for metabolism, induction, efflux transporter based drug-drug interactions and hepatotoxicity. Therefore, we assessed the hepatobiliary disposition for the four ERAs in sandwich-cultured hepatocytes. In general, ambrisentan, bosentan, sitaxsentan, and macitentan demonstrated varying degrees of hepatocyte uptake and accumulation with relatively minimal biliary efflux as determined by the biliary excretion index (BEI) and Cl_b_ (data not shown). The hepatocyte accumulation for each of the 4 ERAs was generally dose dependent (Figure 4). When expressed relative to the extracellular test concentration, the accumulation values were approximately 2x, 20x, 40x and >100x for ambrisentan, bosentan, sitaxsentan and macitentan, respectively (Figure 4). For the ERA concentrations evaluated, both sitaxsentan (100 µM) and macitentan (1, 10 and 100 µM) demonstrated significantly (P<0.05) higher intracellular accumulation values than ambrisentan at the same test concentration (Figure 4).

**Figure 5:**
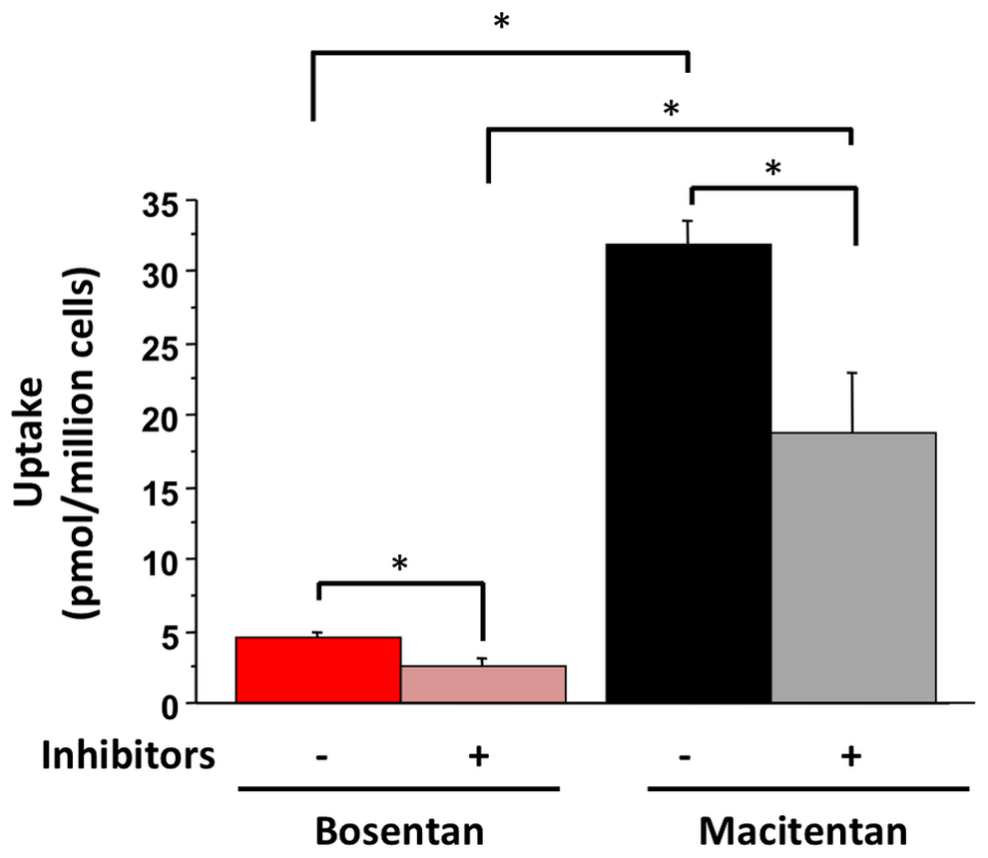
Uptake of bosentan and macitentan into human hepatocytes. ERAs were evaluated either in the absence or presence of the transporter inhibitors rifampicin (40 µM) and cyclosporin A (5 µM). Data presented as mean (±SD) pmol/million cells; n = 4 donors; *P<0.05 for comparisons indicated.

A recent paper reported that macitentan is not a substrate for OATP transporters and that coadministration with cyclosporin A only resulted in a relatively modest effect on macitentan plasma pharmacokinetics in patients (10% increase in exposure and 38% increase in trough concentration) [36]. To further characterize the hepatic uptake of macitentan and bosentan, the effect of transport inhibitors (40 µM rifampicin plus 5 µM cyclosporin A) on the uptake of these ERAs into fresh primary human hepatocytes was assessed. In the absence of transport inhibitors, the cellular uptake of macitentan (31.9±3.4 pmol/million cells) was significantly greater than that of bosentan (4.6±0.7 pmol/million cells; P<0.05) (Figure 5). In the presence of transport inhibitors, the cellular uptake of macitentan and bosentan was reduced by 41% and 46%, respectively (P<0.05) (Figure 5).

### Effect of the ERAs on Endogenous Bile Acids

The liver is not only an important site of bile acid recirculation from portal blood flow into the intestine but also the biosynthesis of bile acids. The accumulation and efflux of the endogenous glycocholic acid (GCA) and glycochenodeoxycholic acid (GCDCA) were determined after 24 h of exposure to the ERAs. There were dose-dependent reductions in the total and cellular accumulation of GCA and GCDCA following ERA treatment, though the magnitude of the reduction varied depending on the ERA (Tables S2 and S3). Generally, macitentan, sitaxsentan, and bosentan demonstrated greater effects on GCA and GCDCA intracellular accumulation and biliary efflux than did ambrisentan. For example, GCA levels in the presence of 10 µM ERA were 75%, 84%, 55% and 32% the values measured in their absence for ambrisentan, sitaxsentan, macitentan and bosentan, respectively (Table S2). Given the 24 h ERA exposure in this assay, the possible influence of ERA metabolite formation on the observed results is unknown.

## Discussion

Drug-induced hepatotoxicity involves the complex interaction of drug metabolic pathways, induction/inhibition of hepatic influx and efflux transporters, and hepatic accumulation of drug and drug metabolites. The canalicular transporter BSEP is responsible for the elimination of monovalent, conjugated bile salts along with some xenobiotics into the bile canaliculi [37]–[39]. In the last few years, BSEP has been identified as a leading mechanistic candidate in the development of drug-induced hepatic toxicity including what is often characterized as mixed hepatitis associated with bosentan, cyclosporin A, rifampicin, troglitazone and glyburide [20]–[22], [25], [26], [40]–[44]. In this study, ambrisentan, bosentan, and macitentan were evaluated for their ability to inhibit key hepatic influx and efflux transporters. Of these, ambrisentan demonstrated the least inhibition (IC_50_) of basolateral expressed OATP1B1 (47.0 µM), OATP1B3 (44.6 µM) and NTCP (>100 µM) while macitentan demonstrated the greatest inhibition (2.0, 2.1 and 9.8 µM for OATP1B1, OATP1B3, and NTCP, respectively). Similarly, macitentan and bosentan inhibited BSEP with IC_50_ values of 11.9 and 42.1 µM, respectively, while ambrisentan had no effect (>100 µM). Overall, ambrisentan, bosentan and macitentan showed relatively minimal inhibitory effects on BCRP, MRP2 and Pgp.

Previously, macitentan has been shown to inhibit murine Pgp (IC_50_ 4.4 µM), canine BCRP (13.2 µM), and human OATP1B1 (4.4 µM) and OATP1B3 (10.0 µM) [45]. Additionally, Bruderer et al. demonstrated very similar IC_50_ values for macitentan with respect to OATP1B1 and OATP1B3 (6.3 µM and 11.8 µM, respectively) [36]. While these prior data are rather comparable to those observed in the current study with respect to OATP1B1 and OATP1B3, the values reported by Weiss et al. for Pgp and BCRP are considerably lower than those seen in the present study (4 µM vs. 64 µM and 13 µM vs. 75 µM, for Pgp and BCRP, respectively). On the other hand, Kim et al. reported no inhibition of human Pgp by macitentan at doses up to 50 µM [46]. While it is difficult to compare across experimental conditions, it is possible that these apparent discrepancies may be at least partially linked to interspecies differences (i.e. human, canine or murine) and/or differences in cell type and probe substrate used. In the assays reported in this study, results for known inhibitors done in side-by-side assay wells were consistent with results in the literature and help to validate the results obtained in the current study.

Given the increased interest in BSEP inhibition as a driving mechanism of hepatotoxicity, it is notable that the observed IC_50_ values for ambrisentan, bosentan and macitentan were >100 µM, 42 µM, and 12 µM, respectively. Of note, under the same assay conditions as used in this study, sitaxsentan was previously found to inhibit BSEP with an IC_50_ of 25 µM, suggesting that macitentan has similar or more potent inhibitory effects on BSEP than either bosentan or sitaxsentan [35]. Though these inhibitory effects of macitentan on BSEP have not been previously reported, the BSEP inhibition values observed in this study for bosentan are comparable to those previously reported (range: 12–76.8 µM) [20], [26], [28], [30]. The results presented here are also in agreement with those of Hartman et al. who demonstrated that bosentan and sitaxsentan inhibited NTCP while ambrisentan did not at the concentrations tested (2, 20, and 100 µM) [29].

The intracellular concentration is the primary driving force for processes that occur inside hepatocytes. Tissue distribution and accumulation is an important factor to take into consideration when assessing the potential for efflux transporter inhibition. When the transport potential for the individual ERAs was evaluated, the intracellular accumulation was the highest for macitentan and the lowest for ambrisentan. In fact, the ambrisentan intracellular accumulation was approximately two times the extracellular dose concentration while that of macitentan was approximately 100 times the extracellular dose concentration. The relative order of accumulation is in agreement with prior studies not including macitentan showing that sitaxsentan accumulated to a greater degree than bosentan or ambrisentan [29].

In human hepatocytes, the cellular uptake of macitentan was approximately 7 times that of bosentan (31.9 pmol/million cells vs. 4.6 pmol/million cells), though we cannot rule out the possibility of some non-specific cellular association with the more lipophilic macitentan. The addition of the transporter inhibitors cyclosporin A and rifampicin significantly reduced the uptake of both macitentan (41% reduction) and bosentan (46% reduction). These data suggest that macitentan is subject to transporter-mediated hepatic uptake. Bruderer et al. showed that over-expressing OATP1B1 or OATP1B3 in CHO cells had no effect on macitentan cellular uptake [36]. While the cause of this apparent discrepancy is unknown, the effects of differences in model systems (i.e. primary human hepatocyte vs. transfected cell-line), contribution of hepatic uptake transporters other than OATPs and assay conditions cannot be excluded. The reduction in bosentan uptake in the presence of transport inhibitors observed here is consistent with prior data showing that bosentan is a substrate for OATP1B1 and OATP1B3, and the data demonstrating that bosentan has clinically relevant drug-drug interactions with both cyclosporin A and rifampicin [47]–[49].

Together, the *in vitro* data presented here suggest that among the ERAs, macitentan is a relatively potent inhibitor of BSEP that readily accumulates in human hepatocytes. However, macitentan is given at a low dose and has correspondingly low plasma exposures relative to other ERAs. This may explain why the reported incidence of serum aminotransferase elevations >3x ULN with 10 mg macitentan were similar to placebo (3.4% vs. 4.5% for macitentan and placebo) following exposure up to 103.9 weeks in the SERAPHIN study [17], [19]. The *in vitro* findings reported in this paper may, however, explain the observation of the apparent incidence of aminotransferase elevations >8x ULN being higher for 10 mg macitentan (2.1%) than placebo (0.4%) [19]. While the clinical implications of this difference are unknown, further data may be needed to accurately assess the long-term risk of drug-induced hepatotoxicity with macitentan.

Together, the data from this study with those of prior studies continue to define the hepatic effects of ERA therapy through the coordinated effects of these compounds on hepatic transport, accumulation and metabolism. This study provides the first direct comparison of ambrisentan, bosentan, sitaxsentan and macitentan with respect to potential mechanisms of clinical hepatoxicity and drug-drug interactions. The results indicate that these ERAs exhibit significant differences in their ability to inhibit key hepatic influx and efflux transporters, with macitentan being the most potent inhibitor followed by bosentan and sitaxsentan. Significant differences were also seen for these ERAs with respect to their cellular accumulation in human hepatocytes and effects on hepatobiliary disposition of exogenous and endogenous bile acids. Macitentan had the highest level of accumulation in hepatocytes and caused the greatest effect on bile acids followed by sitaxsentan and bosentan. Ambrisentan showed very low accumulation and potential to affect hepatic transporters. Further investigation is warranted to continue to define the specific mechanisms by which ERA therapy for the treatment of PAH can induce hepatic injury and to identify important biological differences across individual ERAs with respect to the development of hepatoxicity.

## Supporting Information

Table S1 **Cellular Assays for Transport Inhibition Studies.**
(DOCX)

Table S2 **The Effect of Ambrisentan, Bosentan, Macitentan and Sitaxsentan on the Distribution of Endogenous Glycocholic Acid.**
(DOCX)

Table S3 **The Effect of Ambrisentan, Bosentan, Macitentan and Sitaxsentan on the Distribution of Endogenous Glycochenodeoxycholic Acid.**
(DOCX)
