# Supplementary material for: Evaluation of the Endothelin Receptor Antagonists Ambrisentan, Bosentan, Macitentan, and Sitaxsentan as Hepatobiliary Transporter Inhibitors and Substrates in Sandwich-Cultured Human Hepatocytes
Source: PLoS One. 2014 Jan 30;9(1):e87548. doi: 10.1371/journal.pone.0087548 (PMC3907537; doi:10.1371/journal.pone.0087548)
Supplement: Table S1 — Cellular Assays for Transport Inhibition Studies. (DOCX) [file pone.0087548.s001.docx]

**Table S1. Cellular Assays for Transport Inhibition Studies.**

| **Transporter** | **Transfected Cells** | **Model Substrate** | **Positive Control** |
| --- | --- | --- | --- |
| OATP1B1 | CHO | Fluo 3 | Rifampicin |
| OATP1B3 | CHO | Fluo 3 | Rifampicin |
| NTCP | CHO | Taurocholate | TCDC |
| BCRP | MDCKII | Hoechst 33342 | Fumitremorgin C |
| BSEP | Sf9 cell membrane vesicles | Taurocholate | Cyclosporin A |
| MRP2 | Sf9 cell membrane vesicles | E_2_17βG | Benzbromarone |
| Pgp | MDCKII | Calcein AM | Cyclosporin A, Verapamil |

CHO, Chinese Hamster Ovary; MDCKII, Madin-Darby Canine Kidney strain II, Sf9, Spodoptera frugiperda ovarian cells; E_2_17 βG, Estradiol-17-beta-glucuronide; TCDC, taurochenodeoxycholic acid.
