# Supplementary material for: Evaluation of the Endothelin Receptor Antagonists Ambrisentan, Bosentan, Macitentan, and Sitaxsentan as Hepatobiliary Transporter Inhibitors and Substrates in Sandwich-Cultured Human Hepatocytes
Source: PLoS One. 2014 Jan 30;9(1):e87548. doi: 10.1371/journal.pone.0087548 (PMC3907537; doi:10.1371/journal.pone.0087548)
Supplement: Table S2 — The Effect of Ambrisentan, Bosentan, Macitentan and Sitaxsentan on the Distribution of Endogenous Glycocholic Acid. (DOCX) [file pone.0087548.s002.docx]

**Table S2**. **The Effect of Ambrisentan, Bosentan, Macitentan and Sitaxsentan on the Distribution of Endogenous Glycocholic Acid**

| **Treatment** | **Concentration** | **Total Accumulation** | **Cellular Accumulation** | **BEI** | **Medium Concentration** |
| --- | --- | --- | --- | --- | --- |
|  | **(µM)** | **(% Control)** | **(% Control)** | **(% Control)** | **(% Control)** |
| Ambrisentan | 1 | 87.2 ± 10.8 | 93.0 ± 9.3 | 90.3 ± 13.5 | 101.0 ± 3.5 |
|  | 10 | 73.0 ± 8.4 | 88.2 ± 15.5 | 75.4 ± 8.6 | 91.3 ± 7.5 |
|  | 100 | 39.1 ± 11.9 | 48.8 ± 10.8 | 65.9 ± 6.8 | 95.7 ± 6.7 |
| Bosentan | 1 | 71.9 ± 12.9 | 78.4 ± 14.5 | 85.9 ± 12.6 | 99.6 ± 2.1 |
|  | 10 | 35.9 ± 5.3 | 55.1 ± 14.4 | 32.3 ± 30.1 | 85.5 ± 6.3 |
|  | 100 | 17.4 ± 6.1 | 25.6 ± 8.4 | 35.4 ± 13.9 | 77.1 ± 10.1 |
| Macitentan | 1 | 84.6 ± 12.6 | 81.7 ± 10.7 | 96.9 ± 17.6 | 101.0 ± 3.3 |
|  | 10 | 46.5 ± 8.3 | 62.7 ± 10.5 | 54.9 ± 10.3 | 88.9 ± 1.8 |
|  | 100 | 24.7 ± 7.8 | 38.1 ± 13.6 | 39.4 ± 12.3 | 52.6 ± 1.2 |
| Sitaxsentan | 1 | 98.6 ± 16.1 | 90.9 ± 6.5 | 100.0 ± 18.3 | 104.0 ± 8.1 |
|  | 10 | 60.3 ± 7.2 | 69.8 ± 11.1 | 84.2 ± 6.0 | 92.8 ± 3.8 |
|  | 100 | 14.4 ± 5.4 | 18.6 ± 6.9 | 61.7 ± 12.2 | 75.0 ± 3.5 |

Data presented as mean ± standard error of % control values, n=3 human livers

BEI = biliary excretion index
