# Supplementary material for: Evaluation of the Endothelin Receptor Antagonists Ambrisentan, Bosentan, Macitentan, and Sitaxsentan as Hepatobiliary Transporter Inhibitors and Substrates in Sandwich-Cultured Human Hepatocytes
Source: PLoS One. 2014 Jan 30;9(1):e87548. doi: 10.1371/journal.pone.0087548 (PMC3907537; doi:10.1371/journal.pone.0087548)
Supplement: Table S3 — The Effect of Ambrisentan, Bosentan, Macitentan and Sitaxsentan on the Distribution of Endogenous Glycochenodeoxycholic Acid. (DOCX) [file pone.0087548.s003.docx]

**Table S3**. **The Effect of Ambrisentan, Bosentan, Macitentan and Sitaxsentan on the Distribution of Endogenous Glycochenodeoxycholic Acid**

| **Treatment** | **Concentration** | **Total Accumulation** | **Cellular Accumulation** | **BEI** | **Medium Concentration** |
| --- | --- | --- | --- | --- | --- |
|  | **(µM)** | **(% Control)** | **(% Control)** | **(% Control)** | **(% Control)** |
| Ambrisentan | 1 | 103.0 ± 8.1 | 104.0 ± 3.0 | 95.1 ± 16.3 | 110.0 ± 7.6 |
|  | 10 | 75.7 ± 8.0 | 83.6 ± 11.6 | 81.3 ± 11.0 | 109.0 ± 16.0 |
|  | 100 | 26.3 ± 5.7 | 27.6 ± 3.4 | 76.0 ± 38.1 | 103.0 ± 2.3 |
| Bosentan | 1 | 86.8 ± 3.5 | 88.0 ± 5.9 | 94.5 ± 12.4 | 114.0 ± 9.9 |
|  | 10 | 47.2 ± 2.3 | 54.8 ± 4.4 | 65.7 ± 21.7 | 109.0 ± 14.7 |
|  | 100 | 25.4 ± 6.0 | 30.3 ± 6.7 | 56.8 ± 11.9 | 126.0 ± 7.9 |
| Macitentan | 1 | 90.0 ± 4.0 | 88.7 ± 0.8 | 98.7 ± 14.8 | 111.0 ± 9.0 |
|  | 10 | 48.9 ± 0.9 | 52.1 ± 2.6 | 89.4 ± 14.2 | 98.7 ± 10.1 |
|  | 100 | 8.38 ± 1.4 | 11.4 ± 3.2 | 37.5 ± 21.9 | 51.8 ± 7.1 |
| Sitaxsentan | 1 | 99.3 ± 11.0 | 89.8 ± 10.8 | 112.0 ± 19.8 | 110.0 ± 9.2 |
|  | 10 | 73.8 ± 10.5 | 79.4 ± 12.4 | 85.7 ± 8.4 | 105.0 ± 11.9 |
|  | 100 | 12.4 ± 3.0 | 14.5 ± 2.7 | 55.5 ± 34.3 | 76.5 ± 8.3 |

Data presented as mean ± standard error of % control values, n=3 human livers

BEI = biliary excretion index
